# Quantification of Right and Left Ventricular Function in Cardiac MR Imaging: Comparison of Semiautomatic and Manual Segmentation Algorithms

**DOI:** 10.3390/diagnostics3020271

**Published:** 2013-04-03

**Authors:** Miguel Souto, Lambert Raul Masip, Miguel Couto, Jorge Juan Suárez-Cuenca, Amparo Martínez, Pablo G. Tahoces, Jose Martin Carreira, Pierre Croisille

**Affiliations:** 1 Department of Radiology, University Hospital Complex of Santiago (CHUS), Santiago de Compostela, A Coruña 15706, Spain; E-Mail: josemartin.carreira@usc.es; 2 Department of Radiology, University of Santiago de Compostela (USC), Santiago de Compostela, A Coruña 15706, Spain; E-Mails: lambertraul.masip@usc.es (L.R.M.); ixem.couto@gmail.com (M.C.); jorgejuan.suarez@usc.es (J.J.S.-C.); 3 Department of Cardiology, University Hospital Complex of Santiago (CHUS), Santiago de Compostela, A Coruña 15706, Spain; E-Mail: mariaamparo.martinez.monzonis@sergas.es; 4 Department of Electronics and Computer Science, University of Santiago de Compostela (USC), Santiago de Compostela, A Coruña 15706, Spain; E-Mail: pablo.tahoces@usc.es; 5 Department of Radiology, Hôpital Nord/Centre Hospital Universitaire (CHU) Saint-Etienne, Université de Lyon, Saint-Etienne 42000, France; E-Mail: croisille@creatis.univ-lyon1.fr

**Keywords:** cardiac cine magnetic resonance imaging (MRI), segmentation, ejection fraction (EF), right ventricular function, left ventricular function

## Abstract

The purpose of this study was to evaluate the performance of a semiautomatic segmentation method for the anatomical and functional assessment of both ventricles from cardiac cine magnetic resonance (MR) examinations, reducing user interaction to a “mouse-click”. Fifty-two patients with cardiovascular diseases were examined using a 1.5-T MR imaging unit. Several parameters of both ventricles, such as end-diastolic volume (EDV), end-systolic volume (ESV) and ejection fraction (EF), were quantified by an experienced operator using the conventional method based on manually-defined contours, as the standard of reference; and a novel semiautomatic segmentation method based on edge detection, iterative thresholding and region growing techniques, for evaluation purposes. No statistically significant differences were found between the two measurement values obtained for each parameter (p > 0.05). Correlation to estimate right ventricular function was good (r > 0.8) and turned out to be excellent (r > 0.9) for the left ventricle (LV). Bland-Altman plots revealed acceptable limits of agreement between the two methods (95%). Our study findings indicate that the proposed technique allows a fast and accurate assessment of both ventricles. However, further improvements are needed to equal results achieved for the right ventricle (RV) using the conventional methodology.

## 1. Introduction

Cardiovascular diseases are the leading cause of mortality in the western hemisphere. The analysis of the right and left, global and segmental ventricular heart function, is routinely performed in clinical practice, because it is a powerful prognostic marker of a variety of cardiovascular diseases. Although ultrasound is the most widely-used image modality, it is limited by the relatively frequent poor acoustic window obtained and its operator dependency. Since its introduction, magnetic resonance (MR) imaging has provided important diagnostic information, excellent visualization of cardiac anatomy, and has proven successful to determine the heart function. Besides, MR imaging is a non-invasive technique using non ionizing radiation [1,2,3,4].

In fact, in clinical practice, cardiac MR imaging with steady-state free precession (SSFP) cine sequences—which involves shorter scan time and higher image quality—emerges as the most precise and reproducible imaging technique for the evaluation of both left and right ventricular anatomy and function [5,6,7,8,9]: cardiac MR provides accurate quantitative information of ventricular size and function, blood flow measurements, myocardial viability, and cardiovascular anatomy; for these reasons, it is also one of the preferred methods of imaging in patients with right ventricular diseases, such as, repaired Fallot’s tetralogy, arrhythmogenic right ventricular dysplasia, anomalous pulmonary venous return and pulmonary hypertension [10,11]. 

However, for the quantification of these parameters, right and left ventricular cavities must be segmented into end-diastolic and end-systolic images by manually tracing the endocardial contours [12]. 

Due to the large amount of data produced, manual segmentation of cardiac cine MR images has become a time-consuming and operator dependent process, that hinders the generalized use of these techniques in everyday clinical practice [12]. To deal with these problems, different strategies have been proposed in the literature, including image-driven methods, probabilistic or statistical models, dynamic programming, fuzzy clustering, snakes or active contours, level-sets and its variations, deformable models, active shape or active appearance models, and graph cut approaches. A comprehensive review of these techniques can be found in [13].

Although promising, fully automated methods are not yet widely available, and manual or semi-automatic methods, including efficient mechanisms for correcting imperfect contours, are still preferred in routine clinical practice. Accordingly, the aim of this work was to reduce user interaction to a mouse-click per patient, by developing and validating a semi-automatic segmentation method for the anatomical and functional assessment of both ventricles from short-axis cardiac cine MR images.

## 2. Materials and Methods

### 2.1. Study Population

The study population consisted of 52 consecutive patients (20 women, mean age: 51.7 ± 20.5, age range: 16–83; 32 men, mean age: 48.4 ± 18.5, age range: 19–76) with the following clinical indications: postoperative Fallot’s tetralogy (6), studies to discard possible arrythmogenic dysplasia (8), Ebstein anomaly (1), anomalous pulmonary venous return with left to right shunt (1), dilated cardiomyopathy (10), hypertrophic cardiomyopathy (8), studies of myocardial ischemia/viability (7), myocarditis (6), and non-compacted myocardiopathy (5). Patients with cardiac arrhythmias or dyspnea, implanted pacemakers or defibrillators, or with claustrophobia, were excluded from the study population. The study was retrospective. In all the cases, echocardiography had been previously performed, and all the patients gave written informed consent prior to cardiac MR imaging examination. The study was carried out in accordance with the guidelines of the local ethics committee: The work was approved by the Local (Galician) Ethic Comitte. Informed Consent was also obtained from all patients.

### 2.2. MR Imaging Protocol

Cardiac studies were performed using a 1.5-T MR imaging unit (Magnetom Symphony, Siemens Medical Solutions, Erlangen, Germany). After obtaining scout images and long-axis ECG-gated SSFP cine view for SA plane locations, a stack of 10–14 contiguous short-axis slices, covering both ventricles from the base to the apex, was acquired during 10 to 12 s end-expiratory breath-holds, using a phased array cardiac coil and an ECG-gated SSFP cine sequence, with the following acquisition parameters: 25 frames per cardiac cycle, TR = 40.5 ms, TE = 1.2 ms, flip angle = 50°, matrix = 156 × 192, FOV = 325 × 400 mm, slice thickness = 6 mm. Each slice of the 10–14 slices was acquired in a separate breath hold. To control patient’s cooperation concerning breath-holding, a breathing belt was fixed at the upper abdomen, just below the ribs. 

### 2.3. Image Analysis

**Figure 1:**
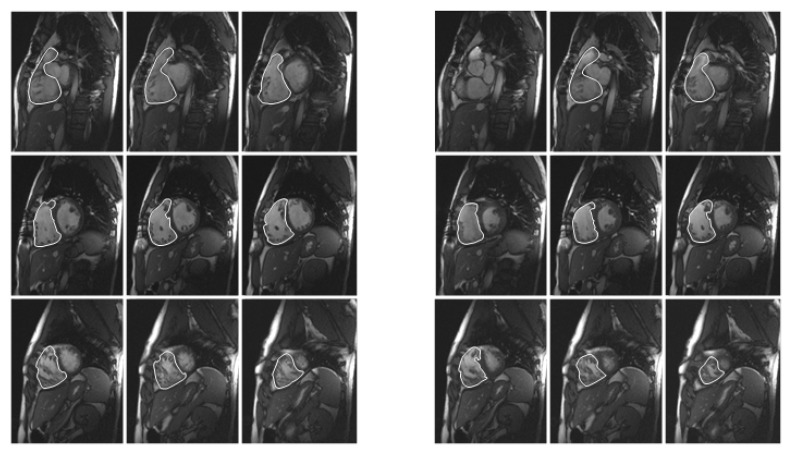
Cardiac magnetic resonance (MR) imaging, trueFISP cine sequence, short-axis views, manual segmentation: end-diastolic (ED; **left**) and end-systolic (ES; **right**) contours of the right ventricle (RV), at the basal, midventricular, and apical levels, in a patient with repaired Fallot’s tetralogy, showing right ventricular dilatation (end-diastolic volume index 148 mL/m^2^); and dysfunction (ejection fraction 32%).

As a preliminary step and to define a same set of images to be used for subsequent segmentation evaluation, ventricular short axis (SA) slices were selected for analysis, beginning with the highest basal slice, as selected from simultaneous display of long-axis and short-axis view, in which at least 50% of the myocardial circumference of the LV was visible in all the cardiac phases. Special attention was given to the right ventricle (RV), so as not to include the atrium [14,15,16,17]. The apical slice was defined as the lowest slice showing intracavitary blood pool. The frames visually showing maximal and minimal ventricular cross-sectional areas at the midventricular level, were considered as end-diastole (ED) and end-systole (ES), respectively. Ventricular contours were traced in every slice, for these two frames, using two segmentation methods (manual and semiautomatic). A difference of one section position was permitted between the most basal slice in ED and ES due to the influence of through plane motion (Figure 1) [4]. Papillary muscles and trabeculae were considered part of ventricular volumes [17]. The end-diastolic volume (EDV) and end-systolic volume (ESV) were calculated by summing up the area enclosed by the endocardium multiplied by the slice thickness, in all the slices imaged at ED and ES, respectively (Simpson’s method). The ejection fraction (EF) was computed as follows: (EDV − ESV)·100/EDV. Function parameters derived from manual contours were computed with the commercial software. Function parameters derived from semi-automatic contours were computed using Simpson’s method.

### 2.4. Manual Method

Ventricular analysis was undertaken on an external workstation (Leonardo, Siemens Medical Solutions, Erlangen, Germany) equipped with a dedicated cardiac post-processing software (Argus, Syngo, Siemens Medical Solutions, Erlangen, Germany) that is routinely used for ventricular volume and function quantification. After manually tracing ventricular contours in a midventricular end-diastolic frame, the contours were propagated throughout the remaining slices imaged at ED. Once checked and corrected, the resulting contours were propagated from ED to ES. Each end-systolic contour was also checked and corrected if required. This segmentation can be considered manual in the sense that the final result was fully under the control of the user. Manual contour tracing of both ventricles was performed for each patient by the radiologist responsible for the study (MS) and, for all patients, segmentation was supervised by a physicist and a computer engineer (LRM and MC) with four years experience in cardiac MRI segmentation, and by a cardiologist (AM) with six years experience in cardiac MRI.

### 2.5. Semiautomatic Method

**Figure 2:**
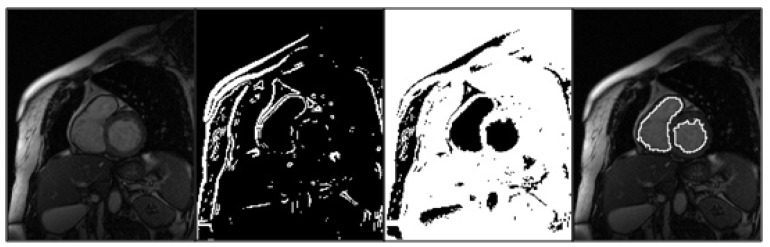
Semiautomatic segmentation scheme showing, from left to right: original gray-scale image, edge detection after iterative thresholding (binary image 1), background overlapping of binary images 1 and 2 (binary image 3), and region growing steps.

Ventricular analysis was also performed on a high performance personal computer (2 Dual-Core AMD Opteron processors 2.80 GHz, 8 GB RAM) with a specifically-designed semiautomatic segmentation method based on edge detection, iterative thresholding and region growing techniques [18]. A brief description of the segmentation scheme is given below (Figure 2).

(i) *Edge detection:* Region boundaries were roughly extracted from the original grayscale image, on the basis of the existing gradient along the contour of an object. These operators are based on the idea that edge information is found by looking at the relationship of a given pixel with its neighbors. In other words, an edge was defined by a discontinuity in grayscale values. Details of implementation of these operators can be found elsewhere [19].

(ii) *Iterative thresholding:* In order to remove the noise from the filtered edge information, a square kernel with a given threshold value k_0_ (0 ≤ k_0_ ≤ 255) was set up automatically in every short-axis view, around the position of the “mouse-click” introduced by the user in a midventricular end-diastolic frame. Then, all the pixels of this kernel were scanned, and the threshold value k_1_ was calculated according to the following expression:

k_1_ = (1/2) × (mean gray-scale below k_0_ + mean gray-scale above k_0_)

If k_0_ ≠ k_1_, k_0_ is updated to k_1_, and k_1_ is recalculated. When the convergence is reached (k_0_ = k_1_), the process finishes, and the algorithm runs through the entire image separating the object from the background pixels, by comparing their intensity with the threshold value (binary image 1). Iterative thresholding is completely automatic, a given threshold value k_0_ (0 ≤ k_0_ ≤ 255) was set up once for all the patients. The obtained result (k_1_) was not sensitive to k_0_. The size of the square kernel can be adjusted empirically, although it was maintained 9 × 9 in the whole study. 

(iii) *Iterative thresholding:* In most of the cases, as a result of the inconsistent edge information contained in the original gray-scale image, there may appear to be discontinuities in the contour of the region of interest (ROI) obtained in the previous step. In order to seal these discontinuities, the thresholding algorithm runs again through the original gray-scale image, as described above (binary image 2).

(iv) *Background overlapping:* Subsequently, an OR logical operation was performed at each pixel location, in order to overlap the backgrounds of the binary images obtained in the previous steps. As a result of the aforementioned operation, a third binary image without any contour discontinuity, was obtained (binary image 3).

(v) *Region growing:* Finally, all the pixels of the square kernel belonging to the object in the third binary image were considered initial seed points. Then the region growing process started, and continued when any of the neighboring pixels belonged to the object. When the process finished, the contour of the ROI was superimposed on the original gray-scale image, and no manual adjustment of generated contours was performed. The region growing algorithm starts and continues when any of the 8-neighboring pixels of every seed point belongs to the object.

### 2.6. Statistical Analysis

For each parameter, the Kolmogorov-Smirnov test rendered a normal data distribution, and the mean ± SD of the results obtained was presented. The relationship between the two measurement values was evaluated using linear regression analysis and Pearson’s correlation coefficient (r), and a two-tailed paired t-test was used to determine the statistical significance (p) of the differences found between them. Our null hypothesis was that no difference exists, and p-values ≤0.05 were considered statistically significant. A 3% change in EF and a 10 mL change in EDV and ESV, was considered of clinical relevance as proposed by Bellenger* et al.* [20]. Agreement between both methods was evaluated through the use of Bland-Altman plots [21] by calculating the bias (mean difference) and the 95% limits of agreement (1.96 SD around the mean difference).

**Table 1:** Quantification of left ventricular parameters using semiautomatic and manual segmentation methods.

	Semiautomatic Mean ± SD	Manual Mean ± SD	Semiautomatic *vs*. Manual r/p *
EDV (mL)	149.2 ± 53.4	153.2 ± 53.7	0.937/0.129
ESV (mL)	69.9 ± 48.4	73.7 ± 48.8	0.961/0.051
EF (%)	56.2 ± 17.7	55.2 ± 15.3	0.918/0.279

EF: ejection fraction; EDV: end-diastolic volume; ESV: end-systolic volume; SD: standard deviation; r: correlation coefficient; (*) p-values ≤ 0.05 were considered statistically significant.

## 3. Results

### 3.1. Quantification of LV Parameters

**Figure 3:**
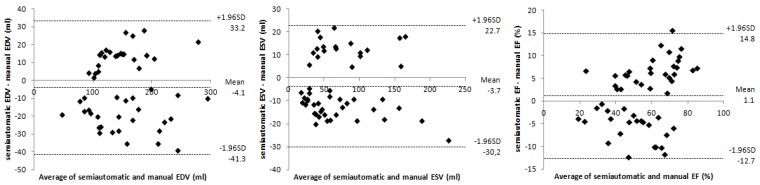
From left to right: Bland-Altman plots show the agreement between semiautomatic and manual segmentation methods, for left ventricular end-diastolic volume (EDV), end-systolic volume (ESV), and ejection fraction (EF).

As shown in Table 1, no statistically significant differences were found for EF and EDV (p > 0.05), and a high correlation value (r > 0.9) was obtained for each parameter. The difference in ESV was close to statistical significance (p ≤ 0.05) but it was not considered clinically relevant (≥10 mL), according to the previously mentioned criteria. The mean difference observed between the parameters using both methods was −4.1 ± 19.0 mL for EDV, −3.7 ± 13.5 mL for ESV, and 1.1 ± 7.0% for EF (Figure 3).

### 3.2. Quantification of RV Parameters

**Figure 4:**
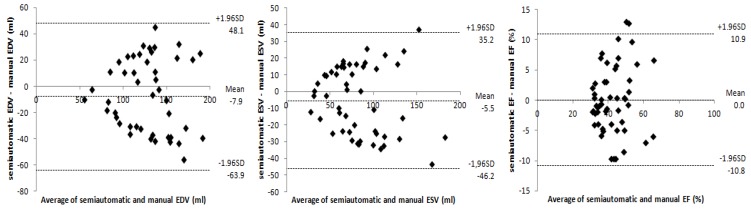
From left to right: Bland-Altman plots show the agreement between semiautomatic and manual segmentation methods, for right ventricular end-diastolic volume (EDV), end-systolic volume (ESV), and ejection fraction (EF).

As shown in Table 2, no statistically significant differences were found for EF and ESV (p > 0.05), and the correlation obtained for each parameter (r > 0.8) was slightly lower than that obtained for the left ventricle. The mean difference observed between the parameters using both methods was −7.9 ± 28.6 mL for EDV, −5.5 ± 20.8 mL for ESV, and 0.0 ± 5.5% for EF (Figure 4).

**Table 2:** Quantification of right ventricular parameters using semiautomatic and manual segmentation methods.

	Semiautomatic Mean ± SD	Manual Mean ± SD	Semiautomatic *vs*. Manual r/p *
EDV (mL)	142.5 ± 62.5	150.4 ± 67.8	0.907/0.050
ESV (mL)	83.5 ± 42.8	89.0 ± 48.2	0.902/0.061
EF (%)	42.6 ± 9.6	42.5 ± 8.9	0.823/0.952

EF: ejection fraction; EDV: end-diastolic volume; ESV: end-systolic volume; SD: standard deviation; r: correlation coefficient; (*) p-values ≤ 0.05 were considered statistically significant.

#### Time Efficiency

The time required to derive ventricular contours was approximately 3 s/slice for the semiautomatic method, and 45–60 s/slice for the manual method, depending on the size of correction needed.

## 4. Discussion

The association of an appropriate cardiac segmentation method and the SSFP imaging sequence provides accurate quantification of the heart function. It is known that, as Kim *et al*. pointed out [16], cardiac cine MR imaging provides excellent tissue contrast between the myocardial muscle and the ventricular cavity, allowing confident edge detection and wall planimetry. However, in clinical practice, the possibility to assess ventricular volumes and function may be hampered not only by the lack of quality of the examinations, but also by the post-processing stages. Heart function MR imaging quantification (the current standard of reference imaging technique) [22] involves prolonged scanning times and multiple apneas; moreover, due to the large amount of data produced, the conventional method, based on manual segmentation of cardiac images, has become a time-consuming and operator dependent process, thus reducing MR imaging clinical usefulness [4]. For this reason, the present work focuses on the development and clinical validation of a semiautomatic segmentation algorithm for the anatomical and functional assessment of both ventricles from cardiac cine MR examinations. Obtained results were compared against those from a commercially available software package based on manually-defined contours, that is routinely used as the standard of reference for ventricular volume and function assessment. 

No relevant differences were observed between the two measurement values, and the results obtained for both ventricles showed a good correlation with data derived from the conventional methodology. Our results are in good agreement with those reported by Bastarrika *et al.* [23], and Mühllenbruch *et al.* [24]: the ESV, EDV and EF obtained by these authors in their studies, after the use of several semiautomatic segmentation algorithms, were well-correlated with those obtained after manual segmentation. These authors applied these methods to the study of the LV in computed tomography. Of interest is that, in our study, an important number of cases with different diseases of the RV were included. However, while our technique succeeds in segmenting the LV (r > 0.9), automated segmentation of the RV (r > 0.8) remains a concern, mainly due to: (1) the complex three-dimensional (3D) shape and prominent trabeculae of the RV; (2) the difficult definition of the valvular plane; and (3) the differences between both methods in the ability to visualize endocardial boundary details and to include papillary muscles and trabeculations in the ventricular cavities. 

Although evaluation of right ventricular function is often overlooked, it is very important for the management of congenital heart malformations; detection of right ventricular dilatation, being of considerable importance, for example, in the indication for surgery in patients with postoperative Fallot’s Tetralogy. There is also an increasing demand for an accurate method to evaluate right ventricular volumes and function in acquired disorders: for example, the functional capacity of the RV offers important prognostic information in pulmonary hypertension [16,25,26,27]. Therefore, a quick reproducible and non-invasive method for the anatomical and functional assessment of the RV would be very useful from a clinical viewpoint.

To overcome the aforementioned limitations, further improvements will be done in future studies. Particularly, our most immediate aim is now improving right ventricular segmentation. Recent studies suggested that by combining long- and short-axis sections in the dataset, the ventricular apex and the basal parts of the LV and, in particular, the RV, can be delineated with more certainty [28]. It is also rather important to provide the method with a high degree of automatism to favour a progressively more routine use. Volumetric 3D analysis should also be investigated [29]. Besides, the population of the study was limited. Thus, the method must be evaluated with a higher number of patients, in particular with right ventricular pathologies. (We have performed our study on a group of patients with a wide range of cardiac diseases. Evaluating right ventricular function, however, should consider that its specific anatomy. A “standard approach” optimized on left ventricular anatomy might cause some measurement bias with regard to right ventricular function, especially in right ventricular diseases). MRI acquisition protocol introduces a minimal inter-slice gap (20% of slice thickness). Unfortunately, this parameter cannot be modified and it does not appear in DICOM header files.

It would also be interesting to evaluate the method on images acquired in a higher magnetic field strength [30] and also to parallel imaging techniques for single breath-hold cine imaging [31]. A comparative analysis of intra- and inter-observer variability of both methods was not included in the present study, because of the identical measurements produced by our semiautomatic segmentation method when applied to the same patient. 

A further limitation of our work is the use of only 4C long-axis cine-SSFP to help in selection of slice planes. The simultaneous use of 2-chambers and 4-chambers long-axis cine-SSFP views to define more accurately the tricuspid plane might improve the accuracy of volume calculation, but requires the acquisition of an imaging plane that is systematically performed and might be missing for retrospective analysis of RV volumes. We allowed a difference of one slice position between the most basal slice in ED and ES, and that might theoretically underestimate the through-plane motion and underestimate the EF, even though our results remain in good agreement with the literature.

Appropriate semiautomatic segmentation of both ventricles was performed in this work. Therefore, the present study demonstrates that the anatomical and functional assessment of both ventricles using the proposed technique is: (1) feasible, reliable and time-effective; (2) reduces the time employed as well as intra- and inter-observer variability to a “mouse click”; (3) does not rely on *a priori* knowledge, providing a true segmentation of the anatomical features present in the image; and therefore (4) can be used in routine clinical practice.

In conclusion, our study findings indicate that the evaluated semiautomatic segmentation method could allow a fast and accurate assessment of the left ventricle, reducing user interaction to a “mouse click” per patient, in order to identify the region of interest (RV, LV, *etc.*). However, further improvements are needed to equal results achieved by manual contour tracing with regard to right ventricular assessment.
